# Fabrication and Evaluation of Bi-layer Tablet Containing Conventional Paracetamol and Modified Release Diclofenac Sodium

**DOI:** 10.4103/0250-474X.65035

**Published:** 2010

**Authors:** M. C. Gohel, R. K. Parikh, S. A. Nagori, B. A. Jethwa

**Affiliations:** Department of Pharmaceutics and Pharmaceutical Technology, L. M. College of Pharmacy, Navrangpura, Ahmedabad-380 009, India

**Keywords:** Bi-layer tablet, diclofenac sodium, factorial design, paracetamol

## Abstract

The objectives of present investigation were to achieve immediate release of paracetamol and tailored release of diclofenac sodium from bi-layer tablets. A 2^3^ full factorial design was adopted using the amount of polyethylene glycol, microcrystalline cellulose and crospovidone as independent variables for fabricating paracetamol tablets. Diclofenac sodium tablets were prepared using hydroxypropyl methylcellulose as a matrixing agent. The results of analysis of variance showed that the friability of paracetamol was distinctly influenced by the formulation variables. The *in vitro* drug release behaviour of diclofenac tablets was compared with a marketed formulation. The optimized formulations of paracetamol and diclofenac sodium were used for manufacturing of bi-layer tablets. The bi-layer tablets showed immediate release of paracetamol and modified release of diclofenac.

Conventional formulations containing paracetamol and diclofenac sodium are commercially available in Indian market. The conventional formulations containing 50 mg of diclofenac sodium are used in arthritis, spondylitis, post-operative pain management and other chronic inflammatory conditions, at a dosing schedule of 3 to 4 times a day. The modified release (MR) tablets of diclofenac sodium containing 100 mg of diclofenac sodium are prescribed as once a day formulations. The major benefits of modified release diclofenac sodium tablets over conventional diclofenac sodium tablets includes reduced dosing frequency and decreased incidence of gastro-intestinal side effects[1–3]. The modified release diclofenac sodium tablets provide analgesic activity for a prolonged duration of time, whereas the combination of conventional paracetamol with MR diclofenac sodium can provide analgesic and anti-pyretic effect. This combination formulation is also beneficial for the patients who are on multiple drug therapy requiring both anti-pyretic and analgesic activity[4]. Dose skipping will be reduced with these types of dosage forms. The objective of the present investigation was to develop a combination dosage form (bi-layer tablet) containing paracetamol and diclofenac sodium.

## MATERIALS AND METHODS

Paracetamol, diclofenac sodium, Cab-O-Sil M5 and microcrystalline cellulose (Avicel PH 102) were received as gift samples from Green Pharmaceuticals (India), Helios Pharmaceuticals Limited (India), Cabot Sanmar Limited (India) and Zydus Cadila (India), respectively. Polyvinylpyrrolidone (PVP K30), crospovidone and hydroxypropylmethylcellulose (HPMC K4M) were received as gift samples from Torrent Pharmaceuticals (India). Polyethylene glycol 6000 (PEG 6000) and magnesium stearate were purchased from S. D. Fine Chemicals Private Limited (India) and Laser Laboratories (India), respectively. Voveran^®^ SR tablets were purchased from a local pharmacy. The other chemicals used were of reagent grade.

### Preparation of microcrystalline cellulose granules:

Microcrystalline cellulose was granulated using 5% w/v aqueous PVP K30 solution. The wet mass was passed through a 20# sieve to obtain granules. The granules were dried at 60° in a tray drier. The 20/40 mesh cut granules were used for preparing paracetamol tablets.

### Preparation of paracetamol tablets:

**Fig. 1:**
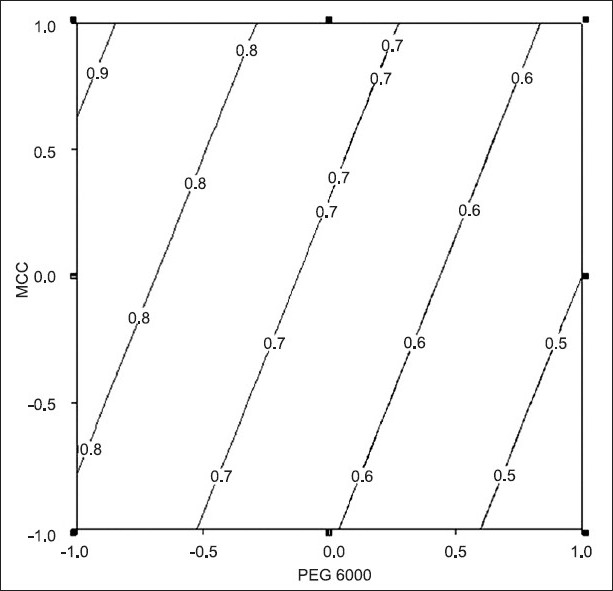
Contour plot for friability of paracetamol tablets Contour plot for friability (––) of paracetamol tablets of batches AF1 to AF8

**Fig. 2:**
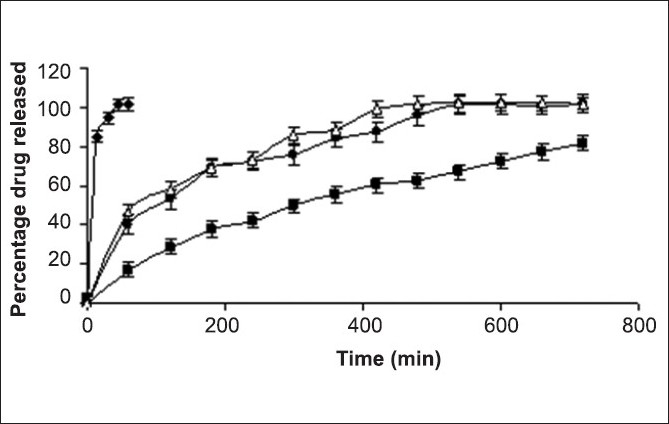
*In vitro* drug release from various formulated batches *In vitro* drug release from various formulated batches AF4 (–♦–), D1 (–■–), D2 (–●–), Voveran^®^ SR (–∆–)

Paracetamol, PEG 6000 and intragranular fraction of crospovidone (half of the total amount of crospovidone) were heated (60±5°) in a porcelain dish. The heated mass was passed through a 20# sieve. The granules were allowed to cool to room temperature and blended with extragranular fraction of granular microcrystalline cellulose (MCC), crospovidone, Cab-O-Sil and magnesium stearate. The tablets were prepared on a single station tablet press (Cadmach Machines Ltd., India). The tablets were evaluated for percentage friability, crushing strength and disintegration time. Tables 1–3 and fig. 1 represents composition and results of the formulated batches. An optimized batch (batch AF4) was subjected for *in vitro* drug release (fig. 2).

**TABLE 1:** FORMULATION AND EVALUATION OF PARACETAMOL TABLETS

Ingredients/Evaluation	Batch code		
	AG1	AG2	AG3
Paracetamol (mg)	500	500	500
PEG 6000 (mg)	100	80	50
Crospovidone (%)^a^	6	6	5
Cab-O-Sil (%)^b^	0.5	0.5	0.5
Magnesium stearate (%)^b^	0.5	0.5	0.5
MCC granules (mg)	110	110	110
Friability (%)	0.3	0.5	2.0
Crushing strength (N)	55	45	25
Disintegration time (min)	11	8	2

a Selected on basis of paracetamol;

b selected on basis of total mass of paracetamol, PEG 6000, crospovidone and MCC granules

**TABLE 2:** FORMULATION AND EVALUATION OF PARACETAMOL TABLETS ACCORDING TO 2^3^ FULL FACTORIAL DESIGN

Ingredients/Results	Batch code							
	AF1	AF2	AF3	AF4	AF5	AF6	AF7	AF8
Paracetamol (mg)	500	500	500	500	500	500	500	500
PEG 6000 (mg)	75	75	75	75	125	125	125	125
Crospovidone^a^ (%)	4	6	4	6	4	6	4	6
Cab-O-Sil (%)^b^	0.5	0.5	0.5	0.5	0.5	0.5	0.5	0.5
Magnesium stearate (%)^b^	0.5	0.5	0.5	0.5	0.5	0.5	0.5	0.5
MCC granules (mg)	62.5	62.5	110	110	62.5	62.5	110	110
Friability (%)	0.8	0.6	0.9	0.8	0.2	0.4	0.4	0.6
Crushing strength (N)	35	75	60	42.5	65.5	70	65	71.3
Disintegration time (min)	23	27	13	7	29	25	20	16

a Selected on basis of paracetamol;

b selected on basis of total mass of paracetamol, PEG 6000, crospovidone and MCC granules

**TABLE 3:** RESULTS OF ANOVA FOR PARACETAMOL TABLETS

Variable	Source of variation	SS	DF	MS	F	Conclusion
Friability	Crospovidone (A)	0.0010	1	0.001013	0.68	NS
	MCC (B)	0.0406	1	0.040613	27.45	Significant
	PEG (C)	0.2556	1	0.255613	172.80	Significant
	AC	0.0630	1	0.063013	42.60	Significant
	Residuals (AB, BC, ABC)	0.0044	3	0.001479		
Crushing strength	Crospovidone (A)	138.6113	1	138.61	0.64	NS
	MCC (B)	5.6112	1	5.61	0.02	NS
	PEG (C)	439.5613	1	439.56	2.06	NS
	Residuals (AB, BC, AC, ABC)	853.095	4	213.27		
Disintegration time	Crospovidone (A)	12.5	1	12.5	1.05	NS
	MCC (B)	288	1	288	24.25	Significant
	PEG (C)	50	1	50	4.21	NS
	Residuals (AB, BC, AC, ABC)	47.5	4	11.87		

The critical value of Fisher (F) value at 5% level of significance is 18.5, SS is sum of squares, DF is degrees of freedom, MS is mean squares, F is Fisher’s variance ratio, NS is non-significant

### Preparation of diclofenac sodium tablets:

Mixture of diclofenac sodium and HPMC K4M (1:1, 1:0.75, 1:0.6, and 1:0.5) were granulated using a blend of water (1 part) and isopropyl alcohol (9 parts). The wet mass was passed through a 20# sieve. The granules were dried at 55° for 15 min in a tray drier. The 20/40 mesh cut granules were used for preparing modified release diclofenac tablets. Cab-O-Sil and magnesium stearate, each at 0.5% w/w, were sequentially mixed with the granules and the tablets were compressed on a single station tablet press (Cadmach Machines Ltd., India). Diclofenac sodium tablets were characterized for percentage friability, crushing strength and *in vitro* drug release. Table 4 and fig. 2 represents the composition and the results of formulated diclofenac sodium tablets.

**TABLE 4:** FORMULATION AND EVALUATION OF BATCHES D1-D4

Ingredients/Evaluation	Batch code			
	D1	D2	D3	D3
Diclofenac sodium (mg)	100	100	100	100
HPMC K4M (mg)	100	75	60	50
Cab-O-Sil (%)^a^	0.5	0.5	0.5	0.5
Magnesium Stearate (%)^a^	0.5	0.5	0.5	0.5
Friability (%)	0.4	0.66	> 1%	> 1%
Crushing strength (N)	49	69	22	10.5

a Selected on basis of total mass of diclofenac sodium and HPMC K4M.

### Preparation of bi-layer tablets:

**Fig. 3:**
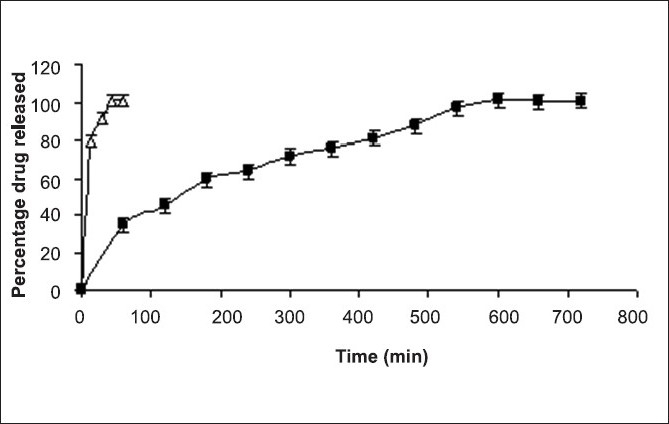
*In vitro* drug release from bi-layered tablets *In vitro* drug release from bi-layered tablets; paracetamol (–∆–), diclofenac sodium (–■–)

For the preparation of bi-layer tablets (batch D5), the granules of the optimized batch of paracetamol (722 mg, batch AF4) were added in the die cavity of single punch tablet machine. The granules of optimized batch of diclofenac sodium (177 mg, batch D2) were added over the granules of paracetamol. The granules were compressed to obtain bi-layer tablets. The tablets were evaluated for percentage friability, crushing strength and *in vitro* drug release (fig. 3).

### Evaluation of granules and tablets:

The angle of repose was measured using the fixed height funnel method[5]. Crushing strength of the tablets was measured using Dr. Scheleuniger tablet hardness tester (Pharmatron 8, Germany) after 24 h of compression (time for stress relaxation). Percentage friability was measured using a friabilator USP (model EF2, Electrolab, India). Twenty tablets were tumbled for 4 min at 25 rpm. The tablets were then dedusted and the loss in weight caused by fracture or abrasion was recorded. Disintegration (DT) of six paracetamol tablets was measured employing an USP (model ED2L, Electrolab, Mumbai). The DT was measured at 37±2° in 900 ml distilled water[6].

*In vitro* drug release of paracetamol, diclofenac sodium tablets and reference product (Voveran^®^ SR) was performed in phosphate buffer (pH 7.4, 37±0.5°) in USP XXIII paddle apparatus (Model TDT-00T, Electrolab, Mumbai, India), at a rotational speed of 50 rpm. Paracetamol and diclofenac sodium contents were estimated by UV/Vis spectroscopic method (Model UV-1700, Pharmaspec, UV/Vis Spectrophotometer, Shimadzu, Japan) at 243 nm and 276 nm, respectively after suitable dilution of the samples. The *in vitro* drug release of paracetamol and diclofenac sodium from bi-layer tablets was measured using HPTLC (Camag, Switerzland)[4]. The peak area was transformed to concentration of drug by reference to a standard calibration curve obtained experimentally (r^2^=0.99). The *in vitro* drug release data of batch D2 were compared with a market formulation (Voveran^®^ SR) and the similarity factor (f_2_) was calculated from the mean drug release data[7]. The method of Bamba *et al*. was adopted to ascertain kinetics of drug release[8]. *In vitro* drug release data of batch D2 were analyzed by different kinetic models in order to evaluate the release mechanism of diclofenac sodium from the matrices[9]. A Fortran software, developed in-house, was used to analyze the data. The least value of sum of square of residuals (SSR) and Fisher's ratio (F) were used to select the most appropriate kinetic model.

### Factorial design:

A three factor, two level full factorial design was adopted for optimization employing the amount of PEG 6000, crospovidone and granules of MCC (extragranular) as independent variables. Friability, crushing strength and disintegration time were selected as dependent variables. In ANOVA, the experimental error (residual) was estimated from the interactions (two or three way). The Fisher's variance ratio (F) was compared with the tabulated value (F_critical_) for grading the factor as significant or nonsignificant. Multiple linear regression was carried out and refined model was evolved (p< 0.05). Further conclusions were drawn using contour plot.

## RESULTS AND DISCUSSION

Crospovidone exhibits high capillary activity and pronounced hydration capacity when it comes in contact with aqueous fluids[10]. Microcrystalline cellulose is a compressible hydrophilic excipient and it also works as an auxiliary disintegrating agent[11]. Hence, granules of MCC were added extragranularly (Table 1). The results shown in Table 1 reveal that the tablets of batches AG1 and AG2 showed acceptable friability (<1%) and crushing strength (>40 N). The disintegration time was less than 11 min due to incorporation of wicking agent (MCC) in the tablets. The disintegration time of paracetamol tablets can be tailored by selecting optimum amount of PEG 6000, MCC and crospovidone. To systematically investigate the influence of the three independent variables (amount of PEG 6000, granular MCC and crospovidone), a 2^3^ full factorial design was adopted. Eight batches (AF1-AF8) containing the same quantity of paracetamol, magnesium stearate and Cab-O-Sil, but varying amounts of PEG 6000, granular MCC and crospovidone were prepared. Table 2 represents composition and results of different batches of paracetamol prepared using factorial design. The high value of independent variables was transformed to +1 and the low value to −1 to facilitate calculations.

Table 2 shows that the percentage friability of tablet of batches AF1-AF8 was within the acceptable limits (<1%). Friability of the tablets ranged from as low as 0.2 to as high as 0.87. Therefore, it can be concluded that at least one of the independent variables or interactions significantly influences friability of the paracetamol tablets. The results of ANOVA following Yates treatment, for friability of paracetamol tablets (batches AF1-AF8) is shown in Table 3. The experimental error (residual) in ANOVA was estimated from the insignificant interactions. The mean squares relating to interactions AB, BC and ABC were distinctly lower than the other mean squares. These interactions were combined to give residual. The results shown in Table 3 reveal that the factors MCC, PEG and interaction between Crospovidone and PEG are significant at p< 0.05. Multiple linear regression analysis was carried out omitting the interaction terms AB, BC and ABC. The main effects A, B and C and the interaction term AC were retained in the model, as they were statistically significant (Eqn. 1), friability=0.5787+0.011A+0.071B–0.178C+0.088AC..(1), (R^2^=0.988, F=60.8, p<0.05)

The effect of the independent variables on friability cannot be judged from the main effects as two-way interaction term (AC) is also significant. Grid search technique or contour plot may be used for evaluating the effect of independent variables on friability. In the present study, contour plot was utilized. The level of crospovidone was fixed at higher level (i.e. +1) to draw contour plot. Fig. 1 reveals that tablets with lower friability were obtained when PEG was used at high level. The result suggests that PEG is a good granulating agent as well as friability arrestor.

The crushing strength of the formulated paracetamol tablets (batches AF1-AF8) ranged from 35 to 75 N. The results of analysis of variance (ANOVA) for crushing strength of paracetamol tablets (batches AF1 to AF8), are shown in Table 3. The mean squares relating to AB, BC, AC and ABC interactions were distinctly lower than the other mean squares. These interactions were combined to give residual. The results shown in Table 3 reveal that none of the factors are significant at 5% and hence multiple linear regression analysis was not carried out.

The disintegration time of the paracetamol tablets (batches AF1-AF8) ranged from 7 to 29 min. The results of analysis of variance (ANOVA) for disintegration time of paracetamol tablets (batches AF1 to AF8) are shown in Table 3. The mean squares relating to the factor AB, BC, AC and ABC were distinctly lower than the other factors. These interactions were combined to give residual. The results shown in Table 3 reveal that MCC exhibited significant effect on disintegration time. Microcrystalline cellulose is known to exhibit disintegration effect due to its hydrophilic nature. The quantity of MCC in paracetamol tablets is far higher than that of crospovidone and hence it exhibited significant effect on disintegration time. Multiple linear regression analysis is not warranted since all terms except MCC were insignificant i.e. p>0.05.

Considering the results of disintegration time, crushing strength and friability, batch AF4 was ranked as the optimized batch and subjected to *in vitro* drug release study. Fig. 2 depicts that more than 85% of paracetamol was released within 15 min from the tablets of batch AF4 and hence, batch AF4 was used for the preparation of bi-layer tablets.

Conventional paracetamol and modified release of diclofenac sodium can be obtained if microspheres of diclofenac and granules of paracetamol are compressed together. Alternatively, bi-layer technology may be adopted. The latter method was chosen in the present study since it is easy to adopt at industry.

Four batches of diclofenac sodium tablets were prepared using varying ratio of drug to HPMC (1:1, 1:0.75, 1:0.6 and 1:0.5). Incomplete drug release (about 82% in 12 h) was observed from tablets of batch D1 (Fig. 2). The drug was released over 12 h from the tablets batch D2 containing 1 part of drug and 0.75 parts of the matrixing agent. Time required to release 90% of drug (t_90_%) was 460 min. Tablets of batches D3 and D4 were not subjected to *in vitro* drug release since the friability was greater than 1%. Hence, considering the results of friability and *in vitro* release, batch D2 was ranked as an optimized batch and used for the preparation of bi-layered tablet. The *in vitro* drug release profile of the tablets of batch D2 was similar to Voveran^®^ SR (f_2_=54). The *in vitro* drug release data of batch D2 was analyzed for establishing kinetics of drug release. The best fit was shown by Korsmeyer-Peppas model with least sum of square of residuals (SSR = 48.7) and Fischer's ratio (F=8.1).

The bi-layer tablets (batch D5) were prepared by using composition of batches AF4 and D2. The bi-layer tablets showed acceptable friability (0.7%) and crushing strength (65 N). High pressure thin layer chromatography (HPTLC) was used to ascertain drug release[4]. The results showed two distinct peaks, one peak of diclofenac sodium at 0.45 R_f_ value and the other of paracetamol at 0.83 R_f_ value. Fig. 3 show that more than 85% of paracetamol was released within 20 min from bi-layered tablet whereas diclofenac sodium was released over 12 h with t_90_% of 495 min.

The bi-layer tablets showed immediate release of paracetamol and modified release (upto 12 h) of diclofenac sodium. The dosage form is patient friendly since the dosing frequency will be reduced. Moreover, the tablets will be easy to swallow, as the tablet weight was less than one gram.
